# The *PTPN22 *1858C/T polymorphism is associated with anti-cyclic citrullinated peptide antibody-positive early rheumatoid arthritis in northern Sweden

**DOI:** 10.1186/ar2214

**Published:** 2007-06-06

**Authors:** Heidi Kokkonen, Martin Johansson, Lena Innala, Erik Jidell, Solbritt Rantapää-Dahlqvist

**Affiliations:** 1 Department of Rheumatology, University Hospital, SE-901 85 Umeå, Sweden; 2 Department of Transfusion Medicine, University Hospital, SE-901 85 Umeå, Sweden

## Abstract

The *PTPN22 *1858C/T polymorphism has been associated with several autoimmune diseases including rheumatoid arthritis (RA). We have shown that carriage of the T variant (CT or TT) of *PTPN22 *in combination with anti-cyclic citrullinated peptide (anti-CCP) antibodies highly increases the odds ratio for developing RA. In the present study we analysed the association between the *PTPN22 *1858C/T polymorphism and early RA in patients from northern Sweden, related the polymorphism to autoantibodies and the HLA-DR shared epitope, and analysed their association with markers for disease activity and progression. The inception cohort includes individuals who also donated samples before disease onset. A case–control study was performed in patients (*n *= 505; 342 females and 163 males) with early RA (mean duration of symptoms = 6.3 months) and in population-based matched controls (*n *= 970) from northern Sweden. Genotyping of the *PTPN22 *1858C/T polymorphism was performed using a TaqMan instrument. HLA-shared epitope alleles were identified using PCR sequence-specific primers. Anti-CCP2 antibodies were determined using enzyme-linked immunoassays. Disease activity (that is, the number of swollen and tender joints, the global visual analogue scale, and the erythrocyte sedimentation rate) was followed on a regular basis (that is, at baseline and after 6, 12, 18 and 24 months). Both the 1858T allele and the carriage of T were associated with RA (χ^2 ^= 23.84, P = 0.000001, odds ratio = 1.69, 95% confidence interval = 1.36–2.11; and χ^2 ^= 22.68, P = 0.000002, odds ratio = 1.79, 95% confidence interval = 1.40–2.29, respectively). Association of the 1858T variant with RA was confined to seropositive disease. Carriage of 1858T and the presence of anti-CCP antibodies was independently associated with disease onset at an earlier age (P < 0.05 and P < 0.01, respectively), while the combination of both resulted in an even earlier age at onset. Smoking was identified as a risk factor independent of the 1858T variant and anti-CCP antibodies.

## Introduction

Recent studies have shown that a missense single nucleotide polymorphism resulting in a substitution of T for C at position 1858 in the protein tyrosine phosphatase nonreceptor type 22 (*PTPN22*) gene is associated with several autoimmune diseases including rheumatoid arthritis (RA) [1-3]. Several of the autoimmune diseases associated with the *PTPN22 *1858T variant are characterized by the presence of autoantibodies. These autoantibodies can be present several years before onset of the disease [4-6]. We have previously shown that a combination of the T variant of *PTPN22 *and anti-cyclic citrullinated peptide (anti-CCP) antibodies in combination strongly predicts the future onset of RA with a specificity of 100% for the disease [7].

The association between the *PTPN22 *polymorphism and RA has been replicated by several groups studying different RA populations [1,8-11]. The first study on *PTPN22 *limited its association to rheumatoid factor-positive disease [1]. This polymorphism has subsequently been associated with both seropositive [8,12] and seronegative disease [13,14].

In addition to genetic factors, environmental factors have been proposed to be of importance in the aetiology of RA. Several studies have suggested smoking to be the major environmental risk factor for RA [15,16]. HLA-shared epitope (SE) alleles and smoking have also recently been shown to act synergistically as risk factors, but only in anti-CCP antibody-positive patients with RA [17].

Considering our findings of a stronger predictive value of the combination of *PTPN22 *1858T variant with anti-CCP antibodies compared with HLA-SE and anti-CCP antibodies for development of RA in individuals before disease onset [7], the aim of the present study was to investigate the 1858 C/T polymorphism in relation to the presence of autoantibodies and HLA-SE alleles in an inception cohort of RA patients from northern Sweden for disease susceptibility, onset and inflammatory activity during the first 2 years. The individuals with blood samples before disease onset were included in the cohort of patients with early RA after disease onset.

## Materials and methods

Patients from the four northern-most counties of Sweden with early RA (duration of symptoms < 12 months) were consecutively included in the study and followed for 2 years. A total of 563 individuals fulfilling at least four of the seven American College of Rheumatology criteria for RA [18] were identified. The patients were assessed clinically at baseline and after 6, 12, 18 and 24 months using the 28-joint count for tender and swollen joints and a global visual analogue scale, and the erythrocyte sedimentation rate was measured. The Disease Activity Score (DAS28) was calculated [19]. Of the patients identified, 505 (342 females, 163 males) were willing to participate and donated DNA for this study. During the study period, 98.0% (*n *= 448/457) of the patients were treated for at least 6 months with disease-modifying antirheumatic drugs and 53.0% (*n *= 244/460) of the patients were receiving prednisolone for at least 6 months. All patients were asked by questionnaire about their smoking habits, and were defined as smoker, nonsmoker ever or previous smoker. Demographic and clinical data of the patients at baseline are presented in Table 1.

**Table 1:** Clinical and demographic data of the rheumatoid arthritis patients at baseline

	Patients (*n *= 505)	Controls (*n *= 970)
Age (years)	54.5 ± 14.0	57.4 ± 11.6
Sex (females/males)	342/163 (67.7/32.3)	705/265 (72.7/27.3)
Symptom duration (months)	6.3 ± 2.8	--
Prednisolone treatment (number receving prednisolone/total number of patients)	195/456 (42.8)	--
Disease-modifying antirheumatic drug treatment (number receving DMARD/total number of patients)	348/403 (86.4)	--
Smoking ever (current, past), (number ever smoking/total number of patients)	321/492 (65.2)	242/899 (26.9)
Anti-cyclic citrullinated peptide antibody-positive (number anti-CCP ab positive/total number of patients)	318/468 (67.9)	5/347 (1.4)
Rheumatoid factor-positive (number RF positive/total number of patients)	264/366 (72.1)	--

Data presented as the mean ± standard deviation or number ratio (percentage).

From among the 505 patients with early RA, a subcohort of 85 individuals was identified as being donors to the Medical Biobank of northern Sweden before disease onset. The median time predating disease onset was 2.7 years (interquartile range = 1.1–6.0 years). Serological analyses before and after disease onset were undertaken for these individuals. The Medical Biobank is population based, and conditions for recruitment into the cohorts and for the collection and storage of blood samples have previously been described in detail [5].

A total of 970 controls from the Medical Biobank of northern Sweden were randomly selected and matched for sex and age within a range of 5 years. The mean age of the controls was 58.2 years (range 25–79 years), and the distribution of males and females was 26.3% and 73.7%, respectively. The Regional Ethics Committee at the University Hospital at Umeå approved this study and all participants gave their written informed consent.

The DNA from patients and controls (*n *= 505 and *n *= 970, respectively) was extracted from ethylenediamine tetraacetic acid-treated whole blood using a standard salting-out method. The 5' nuclease assay was used to determine the *PTPN22 *1858 C/T polymorphism (single nucleotide number rs2476601) as previously described [7]. The PCRs were performed according to the manufacturers' instructions, and detection of the different genotypes was made using an ABI PRISM^® ^7900 HT Sequence Detector System (Applied Biosystems, Foster City, CA, USA). Data were processed using the SDS 2.1 software (Applied Biosystems). The different genotypes were verified by comparison with control samples of known genotype. Genotyping was successful for 504 of the patients.

HLA-DRB1 genotyping was performed using PCR sequence-specific primers from the DR low-resolution kit and DRB1*04 subtyping kits (Olerup SSP AB, Saltsjöbaden, Sweden) according to the previously described method [20]. The HLA-SE genes were defined as DRB1*0404 and DRB1*0401. Results of HLA-DRB1 genotyping were available for 500 patients and 170 randomly selected controls (mean age 53.0 ± 8.9 years, 74.7% females and 25.3% males).

Anti-CCP2 antibodies (*n *= 468 patients) were determined using the Diastat kit from Axis-Shield Diagnostics (Dundee, UK) (cutoff value = 5 units/ml). Rheumatoid factor of the IgM isotype was determined using the agglutination test with sentized sheep erythrocytes as originally described according to Waaler-Rose (*n *= 366).

The chi-square test was used for testing categorical data between groups. Student's *t*-test for independent samples was used to analyse continuous data. Binary logistic regression models were used to estimate predictive values of the 1858T variant of *PTPN22*, HLA-SE alleles, anti-CCP antibodies and smoking. Odds ratios (ORs) were calculated with 95% confidence intervals (CIs). All *P *values refer to a two-sided test, and *P *≤ 0.05 was considered statistically significant. To correct for multiple comparisons, a corrected *P *value (*P*_c_) is also presented. The calculations were performed using the SPSS package (SPSS for Windows 14.0; SPSS Inc., Chicago, IL, USA). The area under the curve using DAS28 values for 24 months was calculated. Any missing values were assumed to be at random; consequently, the last value forward was used for missing DAS28 values at specific time points.

## Results

The genotype distribution of the *PTPN22 *1858C/T polymorphism was in Hardy–Weinberg equilibrium in both the patient group and the control group. The genotype distributions differed significantly between patients and controls (χ^2 ^= 23.85, *P *= 0.000007). The 1858T allele of *PTPN22 *was significantly higher in patients compared with controls, being 18.0% versus 11.4%, respectively (χ^2 ^= 23.84, 2 degrees of freedom, *P *= 0.000001, *P*_c _= 0.000005, OR = 1.69, 95% CI = 1.36–2.11). The CT and TT genotypes were present in 33.0% of the patients and in 21.5% of the controls (χ^2 ^= 22.68, *P *= 0.000002, *P*_c _= 0.00001, OR = 1.79, 95% CI = 1.40–2.29). The CC genotype was significantly decreased in patients compared with controls, at 67.1% versus 78.5%, respectively (χ^2 ^= 22.68, *P *= 0.000002, *P*_c _= 0.00001, OR = 0.56, 95% CI = 0.44–0.72) (Table 2).

**Table 2:** Frequency distribution of the 1858 C/T polymorphism of *PTPN22 *in patients with early rheumatoid arthritis and matched controls

Genotype	Patients (*n *= 504)		Controls (*n *= 970)		χ^2^	*P *value	*P*_c _value	Odds ratio	95% confidence interval
						
	*n*	%	*n*	%					
CC	338	67.1	761	78.5	22.68	0.000002	0.00001	0.56	0.44–0.72
CT	151	30.0	196	20.2	17.53	0.000028	0.00014	1.69	1.31–2.18
TT	15	3.0	13	1.3	4.76	0.02907	0.14535	2.26	1.01–5.08
CT + TT	166	33.0	209	21.5	22.68	0.000002	0.00001	1.79	1.40–2.29
T allele	181	18.0	222	11.4	23.84	0.000001	0.000005	1.69	1.36–2.11

*P*_c_, corrected *P *value.

When the patients were stratified according to autoantibody status, the risk for RA in patients carrying the 1858T variant was confined to those seropositive for anti-CCP antibodies or rheumatoid factor (*P *= 0.0000006, *P*_c _= 0.0000024 and *P *= 0.0000007, *P*_c _= 0.0000028, respectively) compared with controls (Table 3). In patients seronegative for these autoantibodies, carriage of the 1858T variant did not increase the risk for RA (*P *= 0.16 and *P *= 0.17, respectively) compared with controls. There were no significant differences in genotype distributions (*P *= 0.14 and *P *= 0.16) or T-allele frequencies (*P *= 0.16 and *P *= 0.17) in patients positive for compared with patients negative for anti-CCP antibodies or rheumatoid factors.

**Table 3:** Comparison of *PTPN22 *1858 genotypes in rheumatoid arthritis patients stratified according to anti-cyclic citrullinated peptide (anti-CCP) antibodies and rheumatoid factor with all controls

	CT + TT genotype, *n *(%)	CC genotype, *n *(%)	Minor allele frequency	Cases versus all controls^a^				
				
				χ^2^	*P *value	*P*_c _value	Odds ratio	95% confidence interval
Anti-CCP antibody-positive	113 (35.5)	205 (64.5)	0.196	24.99	0.0000006	0.0000024	2.01	1.51–2.67
Anti-CCP antibody-negative	40 (26.7)	110 (73.3)	0.145	1.97	0.16046	0.64184	1.32	0.88–2.00
Rheumatoid factor-positive	96 (36.4)	168 (63.6)	0.199	24.49	0.0000007	0.0000028	2.08	1.53–2.82
Rheumatoid factor-negative	28 (27.5)	74 (72.5)	0.142	1.87	0.17164	0.68656	1.38	0.85–2.23

*P*_c_, corrected *P *value. ^a^Controls presented in Table 2

No significant difference in disease activity (DAS28) calculated by the area under the curve using DAS28 values for 24 months was detected between 1858T carriers and noncarriers (*P *= 0.312). Carriers of the 1858T variant, however, had significantly higher DAS at 6 months (*P *< 0.05, *P*_c _= 0.20). Treatment of the disease at 6 months with disease-modifying antirheumatic drugs or prednisolone was similar in patients with or without the 1858T variant (namely, 87% and 85%, respectively, receiving disease-modifying antirheumatic drugs; and 41% and 44%, respectively, receiving prednisolone).

The mean age at disease onset in patients carrying the 1858T variant (*n *= 166) was 3.0 years less than that in patients lacking this allele (*n *= 338) (T carrier, 52.5 years; and non-T carrier, 55.5 years; *P *< 0.05). Patients with anti-CCP antibodies (*n *= 318) had an earlier onset of the disease compared with patients without these autoantibodies (*n *= 150) (53.2 years for patients with anti-CCP antibodies and 56.8 years for patients without anti-CCP antibodies, *P *< 0.01). A combination of the 1858T variant and anti-CCP antibodies contributed to an even earlier age of disease onset (1858T + anti-CCP antibodies, 51.5 years; compared with 55.2 years for those without this combination; *P *< 0.05).

The HLA-SE alleles were present in 282 (56.4%) of the patients and in 66 (38.8%) of the controls. There was a weak significant association of HLA-SE with the 1858T variant of *PTPN22 *(χ^2 ^= 4.40, *P *= 0.036), with a concordance rate of 52%. The OR for RA in individuals with HLA-SE was 2.04 (95% CI = 1.43–2.91). Carriage of HLA-SE was significantly associated with the presence of anti-CCP antibodies (χ^2 ^= 35.54, *P *< 0.0001).

Anti-CCP antibodies were the strongest risk factor for development of RA in simple logistic regression analyses of the *PTPN22 *1858T variant, HLA-SE allele, anti-CCP antibodies and smoking, whereas the other risk factors had a rather similar OR for RA (Table 4).

**Table 4:** Simple logistic regression analyses determining the odds ratios for different risk factors for rheumatoid arthritis

	Odds ratio	95% confidence interval
Anti-cyclic citrullinated peptide antibodies	145.00	58.72–358.09
Smoking	5.10	4.02–6.46
HLA-shared epitope	2.04	1.43–2.91
T-carriage	1.79	1.41–2.28

Simple logistic regression analyses of the 1858T variant, HLA-SE and smoking after stratification for anti-CCP antibodies showed that smoking was a risk factor for RA in both the anti-CCPantibody seropositive group and the anti-CCP antibody-seronegative group (OR = 6.15, 95% CI = 4.68–8.07 and OR = 3.51, 95% CI = 2.46–5.01, respectively). The 1858T variant and HLA-SE were risk factors for RA only in the anti-CCP antibody-positive group (OR = 2.00, 95% CI = 1.52–2.65 and OR = 3.11, 95% CI = 2.11–4.58, respectively). In multiple regression analysis stratified for anti-CCP antibody and with RA as the dependent variable, the ORs were fairly equal for smoking (OR = 2.64, 95% CI = 1.71–4.08), for HLA-SE (OR = 3.34, 95% CI = 2.18–5.15) and for carriage of the 1858T variant (OR = 2.57, 95% CI = 1.54–4.29) in anti-CCP antibody-positive patients. In anti-CCP antibody-negative patients none of the variables remained significant.

Smoking was a risk factor for RA independent of anti-CCP antibodies and carriage of 1858T in analyses with different combinations of anti-CCP antibodies, carriage of the T allele and smoking compared with nonsmokers without the 1858T variant (Table 5). HLA-SE was a risk factor for RA only in anti-CCP antibody-positive patients; the relative risk of which was further increased in smokers compared with nonsmokers without HLA-SE (Table 6).

**Table 5:** Relative risk for developing rheumatoid arthritis in patients, stratified for anti-cyclic citrullinated peptide (anti-CCP) antibodies

	Non-T carrier (CC)		T carrier (CT, TT)	
	
	Cases/controls	Relative risk (95% confidence interval)	Cases/controls	Relative risk (95% confidence interval)
Anti-CCP antibody-positive				
Smoking-negative	59/511	1.00	37/146	2.17 (1.41–3.35)
Smoking-positive	142/192	6.29 (4.51–8.77)	73/50	12.54 (8.11–19.40)
Anti-CCP antibody-negative				
Smoking-negative	47/511	1.00	17/146	1.36 (0.80–2.33)
Smoking-positive	60/192	3.71 (2.51–5.46)	23/50	5.87 (3.46–9.95)

The combination of carriage or not of the 1858T variant of the *PTPN22 *gene and ever or never smoking were compared for groups stratified for anti-CCP antibodies.

**Table 6:** Relative risk for developing rheumatoid arthritis in patients, stratified for anti-cyclic citrullinated peptide (anti-CCP) antibodies

	Nonsmoking		Smoking	
	
	Cases/controls	Relative risk (95% confidence interval)	Cases/controls	Relative risk (95% confidence interval)
Anti-CCP antibody-positive				
HLA-shared epitope-positive	33/41	1.00	71/49	1.67 (0.95–2.94)
HLA-shared epitope-negative	62/33	2.05 (1.12–3.75)	143/21	7.61 (4.05–14.30)
Anti-CCP antibody-negative				
HLA-shared epitope-positive	40/41	1.00	52/49	1.11 (0.63–1.95)
HLA-shared epitope-negative	23/33	0.71 (0.37–1.38)	31/21	1.92 (0.98–3.77)

The combination of carriage of HLA-shared epitope alleles or not, and ever or never smoking were compared for groups stratified for anti-CCP antibodies.

To analyse the relationship of different factors to anti-CCP antibodies as the dependent variable, multiple logistic regression analysis was performed with the variables 1858T variant, HLA-SE allele and smoking. The 1858T variant was not found to be significant (OR = 1.42 95% CI = 0.90–2.23). Both smoking and HLA-SE predicted significantly the presence of anti-CCP antibodies, with the strongest impact from HLA-SE (OR = 1.69, 95% CI = 1.11–2.57 and OR = 3.25, 95% CI = 2.15–4.92, respectively).

There was no difference in the levels of anti-CCP antibodies in patients with or without the 1858T variant or carrying HLA-SE. In the subcohort of individuals for whom samples were available before disease onset (*n *= 85), anti-CCP antibodies were present in 32.1%. Among those who lacked anti-CCP antibodies before disease onset but who turned anti-CCP antibody positive at disease onset, 62.5% were HLA-SE positive while 40% were HLA-SE negative (not significant). There was no relationship between carriage of the T variant and developing anti-CCP antibodies at disease onset compared with not carrying the T variant (51.4% versus 52.4%, respectively).

## Discussion

In the present study, an association of the 1858T allele of *PTPN22 *with RA was found in 505 individuals from the four northern-most counties of Sweden with early RA,. In agreement with a study from southern Sweden and also other studies, this association was confined to patients with seropositive disease [1,8,12].

Both the 1858T variant and HLA-SE alleles predicted RA, with an increase of the relative risk of each when present in combination with anti-CCP antibodies. This was also found in individuals who subsequently developed RA [7,20]. In the present study, in patients with early RA (that is, after disease onset), there was no significant association between the 1858T variant and anti-CCP antibodies, whereas HLA-SE was strongly associated with anti-CCP antibodies. The increase in the number of anti-CCP antibody-positive patients after disease onset was greater among those carrying HLA-SE alleles. This was evaluated in a subcohort of individuals who were anti-CCP-negative prior to onset of symptoms of disease but who became seropositive after disease onset; however, the number of individuals concerned was small and the difference did not reach statistical significance. These results could suggest that the 1858T variant is important before the disease has developed to a clinical stage, whereas HLA-SE is of significant importance when the disease has developed. Both these and our previous studies confirm that anti-CCP antibodies are the major predictor for RA. Taken together, these results could indicate that both genes contribute to the development of a disease, such as RA, by interfering in separate ways or at separate stages of pathogenesis (for example, by acting at various levels in the immunological process leading to the development of a disease). HLA-SE is believed to be involved in the antigen-presenting process whereas the role of the *PTPN22 *polymorphism for various parts of the immune system is not yet clear. The PTPN22 polymorphism is associated with several other autoimmune diseases, while HLA-SE is strongly related only to RA.

In contrast to most previous studies, we found that the PTPN22 1858T variant and HLA-SE are significantly associated [8,10,12,14,21]. One study has observed an association of the *PTPN22 *1858T variant and HLA-SE, but this was restricted to male patients [13].

Smoking has been shown to be the major environmental risk factor for RA [15,16]. In our study, smoking was a risk factor for RA independent of anti-CCP antibodies and the 1858T variant. In simple logistic regression analyses of anti-CCP antibody-negative patients, smoking was the only significant risk factor for RA, but this significance was lost in multiple logistic regression analysis. In the presence of anti-CCP antibodies, HLA-SE was a risk factor for RA; addition of smoking increased this risk approximately three-fold, which confirms the results presented in a previous study [17].

The genotype and allele frequencies of the *PTPN22 *polymorphism in patients and controls were higher in our study than those reported by some other groups [1,10,14,22]. Our results are consistent with the previously reported increasing south to north gradient in the frequency of the 1858T variant in white European populations [23].

A gene-dosage effect of the 1858T allele has been suggested because a higher susceptibility for RA was found in individuals homozygous for the T allele [8,13]. We similarly found the OR for homozygous carriers of the T allele to be higher than that for heterozygous carriers (2.26 versus 1.69), suggesting a dose effect of the T allele of PTPN22. The number of homozygous carriers we were able to study, however, was small (*n *= 15).

Little is known about the effect of 1858T on disease severity and activity. Most studies investigating the effect of 1858T on clinical characteristics have found no association of 1858T with disease severity or activity [13,21,22]. To analyse the effect of the 1858T variant on disease activity we calculated the area under the curve using DAS28 values for 24 months, but no significant association of 1858T with disease activity was found. There was a significant difference in disease activity at 6 months (*P *< 0.05),, when 1858T carriers had a higher DAS28 than patients without the T variant. This difference was not due to a difference in treatment prescribed; instead, it is possible that carriers of 1858T do not respond primarily as well to the treatment as non-T-carriers. The statistical significance did not remain, however, after correction for the number of performed tests.

It has been shown that the 1858T variant contributes to an earlier age at onset of the disease [12,13]. We were able to confirm this finding; patients with the T variant were significantly younger compared with patients not carrying the T variant. The presence of anti-CCP antibodies, particularly in combination with 1858T, contributed to an earlier age at onset of the disease.

## Conclusion

The PTPN22 1858C/T polymorphism was shown to be associated with early RA in the northern Swedish population and was confined to seropositive disease. Patients carrying the 1858T variant had an earlier onset of the disease, particularly when in combination with anti-CCP antibodies. Furthermore, we could verify a south–north gradient with high frequencies of the *PTPN22 *1858 T variant in patients and controls. Smoking was, in this patient cohort, a risk factor for RA independent of the 1858T variant and seropositivity for anti-CCP antibodies.

## Abbreviations

anti-CCP = anti-cyclic citrullinated peptide; CI = confidence interval; DAS28 = disease activity score; HLA = human leukocyte antigen; OR = odds ratio; PCR = polymerase chain reaction; PTPN22 = protein tyrosine phosphatase nonreceptor type 22; RA = rheumatoid arthritis; SE = shared epitope.

## Competing interests

The authors declare that they have no competing interests.

## Authors' contributions

HK was the main investigator, carried out the genotyping and the statistics, and contributed to preparation of the manuscript. MJ and LI participated in the collection of the material and registration of the patient data. EJ was responsible for the HLA typing. SR-D is the principal investigator, responsible for the Biobank samples, designed the investigation, and participated in data collection, statistical analysis and drafting of the manuscript.
